# Optimization of *Bacillus subtilis* NRC1 growth conditions using response surface methodology for sustainable biosynthesis of gold nanoparticles

**DOI:** 10.1038/s41598-022-25324-w

**Published:** 2022-12-03

**Authors:** Magda A. El-Bendary, Salwa S. Afifi, Maysa E. Moharam, Mostafa M. Abo Elsoud, Noha A. Gawdat

**Affiliations:** 1 Department of Microbial Chemistry, Biotechnology Research Institute, National Research Centre, Giza, Egypt; 2 Department of Microbiology and Immunology, Faculty of Pharmacy for Girls, Al-Azhar University, Cairo, Egypt; 3 Department of Microbial Biotechnology, Biotechnology Research Institute, National Research Centre, Giza, Egypt

**Keywords:** Biological techniques, Biotechnology

## Abstract

Gold nanoparticles (AuNPs) have different unique properties and a wide range of applications in different fields. Thereby, there is a growing urgency for the production of AuNPs using a safe and an economic method. In this study, optimization of fermentation conditions by *Bacillus subtilis* NRC1 for extracellular AuNPs synthesis using response surface methodology was achieved. The data obtained from Plackett–Burman design followed by Box–Behnken design indicated the accuracy and reliability of the model and it could be used to navigate the design space with a reasonable accuracy. Numerical optimization of *Bacillus subtilis* NRC1 active extracellular filtrate production, showed the optimum conditions of 0.74% (w/v) casein hydrolysate, 3.99% (w/v) dextrin, 47 × 10^6^ CFU/ml inoculum size at pH 7.76 and 25 $^\circ$C to give the maximum AuNPs biosynthesis. The model was highly valid and the obtained data had a confidence factor of 98.48%. Statistical optimization resulted in a 2.6-fold increase in AuNPs production compared with that of the non-optimized medium.

## Introduction

In the recent years, nanoparticles (NPs) have attracted a great attention in different applications in medicine, agriculture, electronics, optics, catalysis and in biosensors. It is very well known that NPs (within the size less than 100 nm) are effectively used for most applications due to similar size to biomolecules and easier penetration^1^.

Gold and its compounds have long been used as medicinal agents throughout the history of civilization with its earliest record dating back to 5000 years ago in Egypt^2^. A wide range of AuNPs have been developed by several physical, chemical and biological routes^3^. Green or biological synthesis of NPs has received an interesting attention as it avoids the use of harmful chemicals and hazardous substances. In addition, the synthesis of NPs is at mild conditions of pressure, temperature and pH and does not require the addition of external reducing, capping and stabilizing agents which minimizes their toxicity and expands their biomedical applications^4–6^.

Many biological candidates such as fungi, bacteria and plants are the excellent choice for an eco-friendly green synthesis of AuNPs^7^. Microbes are regarded as potent green nanofactories. They have been used for the synthesis of NPs due to their ease of handling, growing in a low-cost medium, maintaining the safety levels, having the potential of adsorbing the metal ions and reducing them into NPs by the enzymes produced by metabolic processes^2,8^. The extracellular NPs synthesis, is preferred over the intracellular NPs synthesis as it is easier for the NPs extraction from the medium^9^.

There are several Bacilli species that have the ability to synthesize AuNPs as *Bacillus niabensis*, *Bacillus subtilis*, *Bacillus licheniformis*, *Bacillus megaterium*, *Bacillus marisflavi*, *Bacillus cereus*, Endophytic *Bacillus cereus*, *Brevibacillus formosus*, *Lysinibacillus* sp. and *Lactobacillus* sp.^3,10–14^.

It was reported that the synthesis of bacterial AuNPs is linked with bioactive molecules derived from bacteria^15^. AuNPs produced by diverse microorganisms perform numerous functions which are associated in many fields of applications in medicine as diagnosis and treatment of cancer, anti-angiogenesis, anti-arthritic, antimalarial agents and others^8^.

The traditional optimization of microbial fermentation has been carried out by monitoring the effect of one variable at a time (OVAT) on an experimental response. One parameter is changed with keeping other variables at constant levels in this technique. Therefore, the interactive effects among the different parameters and the complete influence of the parameter on the response could not be detected. Besides, a large number of experiments is necessary to conduct the research with this technique and subsequently more required time and expenses. Response surface methodology statistical analysis (RSM) is a well-known statistical analysis method which is applied in the optimization of medium constituents and other critical factors responsible for the production of microbial biomolecules^16,17^. Therefore, RSM could reduce time and costs with more precise results^18^. Also, RSM analyzes the interactions of all factors in a process and explains the prediction of optimum conditions^19^.

The cytotoxic activity of biogenic AuNPs against different types of carcinoma was reported in the literature. Virmani et al.^20^ compared the anticancer behavior of the biogenic AuNPs and chemically synthesized AuNPs towards the cancerous (HeLa, MCF-7, A549 and H1299) and normal (HEK293) cell lines. Cell viability assay suggested that the biologically synthesized particles significantly inhibited the growth of cancer cells in a dose dependent manner, with an IC_50_ value of about 200 µg/ml and were found to be non-toxic towards the normal cells. Barabadi et al.^21^,^21^ reviewed the anticancer activities of biologically synthesized AuNPs against different cancer cell lines and they concluded that biologically synthesized AuNPs had anticancer activities against most of the tested cancer cell lines. In addition, Zarepour et al.^23^ reported the importance of biogenic AuNPs for leukemia therapy. The proposed molecular mechanisms of biogenic AuNPs-induced cytotoxicity in all studies involved over generation of reactive oxygen species and activation of apoptotic pathways resulting in a cell death.

In a previous study the authors synthesized AuNPs using extracellular filtrate of isolated *Bacillus subtilis* NRC1, characterized the produced AuNPs using different physical methods, and studied their biological activities with their application as liver anti- fibrosis agent (under publication). *Bacillus subtilis* AuNPs were characterized using UV–visible spectroscopy which showed a surface plasmon resonance (SPR) spectrum at 540–550 nm. Energy dispersive X-ray spectroscopy (EDX) peak of AuNPs was shown at 2 keV, which confirmed the presence of elemental Au. High resolution transmission electron microscopy (HRTEM) figures of AuNPs showed well dispersed particles with spherical, hexagonal and oval shapes, with the size of 4.7–25 nm. Selected area electron diffraction pattern (SAED) for a single particle showed ring pattern with very fine spots indicating the polycrystallinity structure of the tested AuNPs. Dynamic light scattering spectroscopy (DLS) study revealed, the average diameter size of the particles was 23 nm. AuNPs were negatively charged as determined by zeta potential measurement and the charges were −25 mV. Fourier transform infrared spectroscopy (FTIR) spectra of AuNPs revealed that they were capped with reducing biomolecules secreted by *Bacillus subtilis*. The spectra strongly indicated the presence of proteins covering them which might play a role in the synthesis and stabilization of NPs.

In the current study, optimization of the growth variables of *Bacillus subtilis* NRC1 for production of AuNPs active extracellular filtrate was studied. Plackett–Burman design followed by Box–Behnken design were applied in this study. Also, the optimum cultural and nutritional conditions for maximum AuNPs production were obtained and validated.

## Material and methods

### Microorganism

The bacterial culture NRC1 was isolated from a golden earring and molecularly identified using 16S rRNA gene sequence as *Bacillus subtilis* NRC1 and it was deposited in NCBI GenBank with an accession number of MK333152^24^. It was used for the green synthesis of AuNPs.

### Biosynthesis of AuNPs

The extracellular bacterial filtrate at an appropriate dilution and 2 mM HAuCl_4_ (1:1) were incubated for 24 h at 30 °C under static and illuminated conditions. The change in solution colour from pale yellow to purple was indicated, as positive AuNPs synthesis.

### UV–vis investigation

The effect of different parameters on the biosynthesis of AgNPs were characterized by absorption spectral scan using double beam UV–vis spectrophotometer (Carry 100 Ultraviolet–visible spectrophotometer, Agilent, USA) at a resolution of 1 nm in a wave length range between 300 and 700 nm to determine their surface plasmon resonance peaks. Under the experimental conditions, both the size and concentrations of nanoparticles may vary and both of these affect the UV–vis spectrum. Therefore, the absorbance intensity was taken as an indication of an increase or decrease in the nanoparticles synthesis.

### Optimization of cultural and nutritional variables for improving AuNPs synthesis using response surface methodology

Different cultural and nutritional independent variables were screened with special focusing on the enhancement of AuNPs synthesis. Primarily, the effects of nitrogen sources, carbon sources and metal ions were screened using one variable at a time (OVAT) method, where each of the variables was amended while other variables being constant, then response surface statistical methodology was used to determine the significance of these variables and interactions with other variables.

### Effect of carbon sources, nitrogen sources and metal ions using OVAT method

#### Effect of nitrogen sources

This experiment was designed to find out the effect of certain nitrogen sources in the medium on the efficiency of bacterial filtrates for AuNPs synthesis^25^. Different nitrogen sources were added individually to 25 ml nutrient broth in 250 ml Erlenmeyer flasks, at a concentration that is equivalent to the nitrogen content in 0.2% of sodium nitrate. These nitrogen sources were sodium nitrite, potassium nitrate, casein hydrolysate, urea, ammonium phosphate, ammonium sulphate, peptone and yeast. Then the media were inoculated with *Bacillus subtilis* NRC1 and incubated for 3 days at 30 $^\circ$C under shaking at 150 rpm.

#### Effect of carbon sources

In this experiment attempts were made to find out the effects of some carbon sources on the growth and biomolecules production for efficient AuNPs synthesis^25^. Individually supplemented nutrient broth medium with 15 carbon sources representing mono, di and poly-saccharides at 1% were added to 25 ml nutrient broth in 250 ml Erlenmeyer flasks. Carbon sources were starch, dextrin, lactose, maltose, sucrose, glucose, fructose, xylose, arabinose, raffinose, mannose, galactose, glycerol, sodium acetate and tripotassium citrate. These flasks were inoculated with *Bacillus subtilis* NRC1 and incubated for 3 days at 30 $^\circ$C under shaking at 150 rpm.

#### Effect of mineral salts

Metal ions were tested for their ability to increase the biomolecules in the bacterial filtrates which were used in AuNPs synthesis^26^. Different mineral salts were added individually at the reported concentrations in the literature to 25 ml nutrient broth in 250 ml Erlenmeyer flasks (calcium sulphate 10^−4^ M, magnesium sulphate 10^−4^ M, sodium molybdate 10^−6^ M, manganese sulphate 10^−4^ M and zinc sulphate 10^−4^ M). These flasks were inoculated with *Bacillus subtilis* NRC1 and incubated for 3 days at 30 $^\circ$C under shaking at 150 rpm.

### Response surface methodology statistical analysis

Optimization of fermentation conditions experiments for AuNPs biosynthesis by *Bacillus subtilis* NRC1 extracellular filtrate was carried out using two designs: Firstly, Plackett–Burman (PBD) followed by Box–Behnken (BBD). RSM designs were constructed using Design – Expert software (Stat-Ease Inc., Minneapolis, MN, USA, ver. 7.0). All experiments were done with 25 ml nutrient broth in 250 ml Erlenmeyer flasks. The flasks were inoculated with *Bacillus subtilis* NRC1 and incubated for 3 days under shaking at 150 rpm and 30 $^\circ$C. The responses in these experiments were absorbance intensities at the maximum peaks that are specific for the biosynthesis of AuNPs. All the experiments were carried out in triplicate and the average values were recorded.

### Plackett Burman design (PBD)

Plackett–Burman experiments were carried out for screening the most significant variables affecting AuNPs synthesis. The PBD was based on six independent variables; (A): casein hydrolysate concentration (%), (B): dextrin concentration (%), (C): initial pH, (D): incubation period (days), (E): inoculum size (CFU × 10^6^/ml) and (F): temperature (°C). This design contained 15 runs in 3 levels with 3 center points as shown in Tables 1 and 2. The responses in these experiments were the absorbance intensities at the maximum peaks that are specific for the biosynthesis of AuNPs.

**Table 1:** Summary of PBD for AuNPs biosynthesis, using *Bacillus subtilis* NRC1 extracellular filtrate.

Independent Factor	Symbol	Units	Levels		
			−1	0	1
Casein hydrolysate	A	%	0.16	0.44	0.72
Dextrin	B	%	1	2.5	4
Initial pH	C	_	6	7	8
Incubation period	D	days	1	3	5
Inoculum size	E	CFU × 10^6^/ml	56	168	280
Temperature	F	$^\circ$C	20	30	40

**Table 2:** PBD layout for AuNPs biosynthesis, using *Bacillus subtilis* NRC1 extracellular filtrate.

Run	Casein hydrolysate		Dextrin		Initial pH		Incubation period		Inoculum size		Temperature		Maximum peak absorbance intensity	
	Coded	Actual (%)	Coded	Actual (%)	Coded	Actual	Coded	Actual (days)	coded	Actual (CFU × 10^6^/ml)	coded	Actual (°C)	Experimental value	Predicted value
**1**	0	0.44	0	2.5	0	7	0	3	0	168	0	30	2.78	2.88
**2**	1	0.72	−1	1	1	8	1	5	−1	56	1	40	0.74	0.77
**3**	−1	0.16	1	4	−1	6	1	5	1	280	−1	20	0.19	0.19
**4**	0	0.44	0	2.5	0	7	0	3	0	168	0	30	3	2.88
**5**	−1	0.16	1	4	1	8	−1	1	1	280	1	40	1.8	1.80
**6**	1	0.72	1	4	−1	6	−1	1	−1	56	1	40	0.91	0.88
**7**	−1	0.16	−1	1	−1	6	−1	1	−1	56	−1	20	0	0.03
**8**	1	0.72	1	4	−1	6	1	5	1	280	1	40	1.14	1.17
**9**	−1	0.16	−1	1	−1	6	1	5	−1	56	1	40	0.53	0.50
**10**	−1	0.16	−1	1	1	8	−1	1	1	280	1	40	1.09	1.09
**11**	−1	0.16	1	4	1	8	1	5	−1	56	−1	20	2.73	2.73
**12**	1	0.72	−1	1	−1	6	−1	1	1	280	−1	20	1.53	1.53
**13**	0	0.44	0	2.5	0	7	0	3	0	168	0	30	2.87	2.88
**14**	1	0.72	1	4	1	8	−1	1	−1	56	−1	20	2.03	2.03
**15**	1	0.72	−1	1	1	8	1	5	1	280	−1	20	0.64	0.61

Plackett Burman design is based on the first order linear equation:

$${\text{Y}} = \beta_{0} + {{\varvec{\Sigma}}}\beta_{i} X_{i}$$

where Y is the predicted response (production of AuNPs), *β*_*0*_ is the model intercept, *β*_*i*_ is the variables estimates and *X*_*i*_ represents the coded independent variable which were coded as A, B , C,…. in this study.

The results of PBD followed by BBD were used for determining the significant variables that were effective for the AuNPs synthesis by the produced filtrate of *Bacillus subtilis* NRC1*.*

### Box–Behnken design (BBD)

Response surface modeling and optimization of AuNPs biosynthesis conditions were accomplished using a quadratic BBD. Through this design, five variables; (A): casein hydrolysate concentration (%), (B): dextrin concentration (%), (C): initial pH, (D): inoculum size (CFU × 10^6^/ml) and (E): temperature (°C), each with three levels comprising forty six runs (Tables 3 and 4). The responses in all experiments were expressed in terms of absorbance intensities of the biosynthesized AuNPs.

**Table 3:** Summary of BBD used for AuNPs biosynthesis, using *Bacillus subtilis* NRC1 extracellular filtrate.

Independent Factor	Symbol	Units	levels		
			−**1**	**0**	**1**
Casein hydrolysate	A	%	0.16	0.72	1.28
Dextrin	B	%	1	2.5	4
Initial pH	C	_	6	7	8
Inoculum size	D	CFU × 10^6^/ml	56	168	280
Temperature	E	$^\circ$C	25	30	35

**Table 4:** BBD used for AuNPs biosynthesis, using *Bacillus subtilis* NRC1 extracellular filtrate.

Run	Casein hydrolysate		Dextrin		Initial pH		Inoculum size		Temperature		Maximum peak absorbance intensity	
	Coded	Actual (%)	Coded	Actual (%)	Coded	Actual	Coded	Actual (CFU × 10^6^/ml)	Coded	Actual (ºC)	Experimental	Predicted
1	0	0.72	−1	1	−1	6	0	168	0	30	1.54	1.38
2	0	0.72	−1	1	0	7	1	280	0	30	0.6	0.66
3	0	0.72	0	2.5	−1	6	0	168	1	35	0.68	0.79
4	1	1.28	1	4	0	7	0	168	0	30	3.1	3.16
5	1	1.28	−1	1	0	7	0	168	0	30	0.2	0.26
6	0	0.72	0	2.5	1	8	0	168	1	35	1.87	1.85
7	0	0.72	1	4	−1	6	0	168	0	30	0.76	0.6
8	−1	0.16	−1	1	0	7	0	168	0	30	1.03	1.09
9	0	0.72	−1	1	0	7	−1	56	0	30	0.88	0.86
10	1	1.28	0	2.5	−1	6	0	168	0	30	2.21	2.09
11	0	0.72	0	2.5	0	7	1	280	−1	25	1.98	1.87
12	0	0.72	0	2.5	0	7	0	168	0	30	1.59	1.71
13	0	0.72	0	2.5	0	7	0	168	0	30	1.71	1.71
14	0	0.72	−1	1	0	7	0	168	1	35	2.19	2.14
15	0	0.72	1	4	0	7	0	168	1	35	2	1.95
16	0	0.72	−1	1	1	8	0	168	0	30	1.08	1.18
17	0	0.72	1	4	0	7	1	280	0	30	2.75	2.81
18	0	0.72	0	2.5	−1	6	−1	56	0	30	0.95	1.16
19	0	0.72	0	2.5	−1	6	1	280	0	30	0.46	0.95
20	0	0.72	0	2.5	0	7	1	280	1	35	1.84	1.53
21	0	0.72	0	2.5	1	8	1	280	0	30	2.01	2.01
22	0	0.72	0	2.5	0	7	0	168	0	30	1.74	1.71
23	0	0.72	1	4	0	7	0	168	−1	25	1.7	1.65
24	1	1.28	0	2.5	0	7	−1	56	0	30	0.5	0.41
25	−1	0.16	0	2.5	−1	6	0	168	0	30	0.37	−0.02
26	0	0.72	1	4	0	7	−1	56	0	30	3.05	3.03
27	0	0.72	0	2.5	0	7	0	168	0	30	1.74	1.71
28	0	0.72	0	2.5	0	7	0	168	0	30	1.97	1.71
29	−1	0.16	0	2.5	0	7	0	168	−1	25	1.25	1.73
30	0	0.72	−1	1	0	7	0	168	−1	25	1.27	1.22
31	0	0.72	0	2.5	1	8	0	168	−1	25	2.51	2.19
32	1	1.28	0	2.5	0	7	0	168	1	35	2.76	2.74
33	0	0.72	0	2.5	0	7	0	168	0	30	1.33	1.71
34	0	0.72	0	2.5	−1	6	0	168	−1	25	1.12	1.13
35	−1	0.16	0	2.5	0	7	1	280	0	30	2.38	2.29
36	−1	0.16	0	2.5	0	7	−1	56	0	30	2.23	2.14
37	1	1.28	0	2.5	0	7	1	280	0	30	2.79	2.69
38	−1	0.16	0	2.5	0	7	0	168	1	35	0.23	0.49
39	1	1.28	0	2.5	1	8	0	168	0	30	2.39	2.39
40	−1	0.16	1	4	0	7	0	168	0	30	2.69	2.75
41	0	0.72	0	2.5	1	8	−1	56	0	30	1.79	2.22
42	0	0.72	1	4	1	8	0	168	0	30	2.81	2.91
43	0	0.72	0	2.5	0	7	−1	56	1	35	1.85	1.75
44	−1	0.16	0	2.5	1	8	0	168	0	30	2.09	1.8
45	1	1.28	0	2.5	0	7	0	168	−1	25	1.99	2.18
46	0	0.72	0	2.5	0	7	−1	56	−1	25	2.39	2.08

The second degree polynomial equation, which includes all interaction terms, was used to calculate the predicted response:

$${\text{Y}}_{{{\text{response}}}} = \beta_{0} + {{\varvec{\Sigma}}}\beta_{i} X_{i} + {{\varvec{\Sigma}}}\beta_{ii} X^{2}_{i} +_{{}} {{\varvec{\Sigma}}}\beta_{ij} X_{i} X_{j}$$

where Y_response_ represents the predicted response (biosynthesis of gold nanoparticles), *β*_*0*_ is the intercept, *β*_*i*_ is the linear coefficient, *βii* signifies the quadratic coefficients while *β*_*ij*_ is the cross product coefficients and *X*_*i*_ , *X*^*2*^_*i*_ , *X*_*j*_ represent the coded independent variables. In the present study the independent variables were coded as A, B and C therefore, the second order polynomial equation was as follows:

$${\text{Y}}_{{{\text{response}}}} = \beta_{0} + \beta_{1} A + \beta_{2} B \, + \beta_{3} C \, + \beta_{12} AB + \beta_{13} AC + \beta_{23} BC \, + \beta_{11} A^{2} + \beta_{22} B^{2} + \beta_{33} C^{2} .$$

Data obtained from both designs were analyzed by analysis of variance (ANOVA) and multiple regression analysis using Design Expert software, Version 7.0 (Stat-Ease Inc. Minneapolis, MN, USA). Value of "Prob > F" less than 0.05 was used as a parameter to indicate the significant model terms. The equations for the model were generated and the interpretation of the data was based on the signs (positive or negative effect on the response) and statistical significance of coefficients (P < 0.05). 3D surface graphs were designed by using Design Expert software to analyze the relationship between the different variables.

### Model validation

In order to determine the precision of the statistical experimental model (BBD) and to obtain the optimum conditions for maximum AuNPs biosynthesis, the independent variables were numerically optimized according to the model using Design Expert software ver. 7.0 where the values of AuNPs biosynthesis were theoretically calculated. The optimum conditions were experimentally applied and the produced response was compared with the theoretical results.

### Nitrate reductase assay

Nitrate reductase activity was tested according to^27^. Potassium nitrate was added to the extracellular bacterial filtrate (enzyme source) and incubated in the dark for 24 h. Then, diazo coupling reagent (1% sulfanilamide in 3 N HCl and 0.02% N-(1-naphthyl) ethylene diamine hydrochloride) was added to the reaction mixture and diluted 10 folds. After incubation for 30 min in dark at 30 °C, optical density was recorded at 540 nm. Nitrate reductase activity was expressed as micromoles of nitrite produced per ml of filtrate per min (µmol /ml/ min).

## Results

### Effect of carbon sources, nitrogen sources and metal ions using OVAT method

#### Effect of medium supplementation with different nitrogen sources on the synthesis of AuNPs

As shown in Table 5, casein hydrolysate gave the highest absorbance intensity (2.20) for AuNPs synthesis. While it was noted that AuNPs synthesis decreased significantly by addition of potassium nitrate, ammonium phosphate and ammonium sulphate in comparison with nutrient broth medium.

**Table 5:** Effect of different (a) carbon sources (1%), (b) nitrogen sources and (c) metal ions on the synthesis of AuNPs using the extracellular filtrate of *Bacillus subtilis* NRC1.

Carbon source	AuNPs synthesis		Nitrogen source	AuNPs synthesis		Mineral salt	AuNPs synthesis	
	Maximum peak wave length	Absorbance intensity		Maximum peak wave length	Absorbance intensity		Maximum peak wave length	Absorbance intensity
Starch	550	1.25 ± 0.05f.	Sodium nitrite	554	1.38 ± 0.06bc	Calcium sulphate	560	1.17 ± 0.04b
Dextrin	544	2.39 ± 0.09a	Potassium nitrate	555	0.55 ± 0.06e	Magnesium sulphate	561	0.51 ± 0.21c
Lactose	551	1.46 ± 0.07de	Casein hydrolysate	547	2.20 ± 0.11a	Sodium molybdate	552	1.15 ± 0.07b
Maltose	548	1.99 ± 0.04b	Urea	550	1.23 ± 0.02 cd	Manganese sulphate	557	1.11 ± 0.07b
Sucrose	–	–	Ammonium phosphate	554	0.76 ± 0.04e	Zinc sulphate	556	1.20 ± 0.05b
Glucose	–	–	Ammonium sulphate	555	0.64 ± 0.02e	Control (NB medium)	549	1.36 ± 0.03a
Fructose	555	1.05 ± 0.05 g	Peptone	547	1.12 ± 0.05d			
Xylose	549	1.52 ± 0.08 cd	Yeast extract	543	1.55 ± 0.08b			
Arabinose	547	2.15 ± 0.12b	Control (NB medium)	549	1.27 ± 0.06 cd			
Raffinose	548	1.02 ± 0.04 g						
Mannose	560	1.61 ± 0.05 cd						
Galactose	547	0.89 ± 0.04 g						
Anhydrous glycerol	–	–						
Sodium acetate	549	0.98 ± 0.05 g						
Tripotassium citrate	556	1.67 ± 0.08c						
Control (NB medium)	556	1.29 ± 0.04ef						

Absorbance is presented as means ± SE. a,b,c………Different letters in the same column are significantly different at P < 0.05.

– No synthesis.

#### Effect of medium supplementation with different carbon sources on the synthesis of AuNPs

The obtained results in Table 5 showed that dextrin gave the highest absorbance intensity (2.39) for AuNPs. Also, maltose and arabinose showed a valuable enhancement for AuNPs synthesis. On the other hand, sucrose, glucose and anhydrous glycerol completely inhibited AuNPs synthesis.

#### Effect of medium supplementation with different metal ions on the synthesis of AuNPs

Table 5 showed that the supplementation of nutrient broth medium with the tested metal ions had a negative impact on the AuNPs synthesis.

### Optimization of *Bacillus subtilis* NRC1 filtrate production for AuNPs synthesis using RSM

#### Screening of the significant variables using Plackett Burman design (PBD) for AuNPs synthesis

Fifteen runs with different combinations of the independent variables were tested and their influences on AuNPs synthesis were shown in Table 2. The data obtained for AuNPs production were statistically analyzed using analysis of variance (ANOVA) and were shown in Table 6. According to these data, the model F-value of 66.78 and *p-*value of 0.0027 referred to the significance of the model. In addition, A, C, D, F, AB, AC, AE and BC were significant model terms (*p-*value less than 0.05). F-value of 726.57 implied a significant model curvature. However, the "Lack of Fit F-value" of 0.54 implied that, the Lack of Fit was not significant relative to the pure error.

**Table 6:** Analysis of variance (ANOVA) of PBD for AuNPs biosynthesis, using the extracellular filtrate of *Bacillus subtilis* NRC1.

Source	Sum of squares	df	Mean square	F-value	*p*-value* prob > F
Model	6.93	10	0.69	66.78	0.0027
A-casein hydrolysate	0.57	1	0.57	54.48	0.0051
B-dextrin	2.689E−003	1	2.689E−003	0.26	0.6458
C-initial pH	0.91	1	0.91	87.81	0.0026
D-incubation period	0.51	1	0.51	48.93	0.0060
E-inoculum size	3.600E−004	1	3.600E−004	0.03	0.8641
F-temperature	0.38	1	0.38	36.34	0.0092
AB	0.23	1	0.23	21.84	0.0185
AC	1.41	1	1.41	136.00	0.0014
AE	0.27	1	0.27	26.21	0.0144
BC	1.08	1	1.08	103.61	0.0020
Curvature	7.54	1	7.54	726.57	0.0001
Residual	0.03	3	0.01		
Lack of fit	0.01	1	0.01	0.54	0.5373
Pure error	0.02	2	0.01		
Cor total	14.50	14			

F value = 66.78; R^2^ = 0.99; Adjusted R^2^ = 0.98.

*: Values of "prob > F" less than 0.05 indicate model terms are significant.

The determination coefficient (R^2^) of the model was 0.99, indicating that there was a very good fit between the observed and predicted values of AuNPs biosynthesis and implied that the model is reliable for this process. The determination coefficient (R^2^) value (is always between 0 and 1) provided a measure of how much variability in the observed response values. The closer the R^2^ value to 1, the stronger the model is and the better it predicts the response^28^. The model R^2^ was 0.99 and the adjusted-R^2^ was calculated to be 0.98, indicating a good agreement between the experimental and predicted values of AuNPs synthesis. The model adequate precision, which measured the signal to noise ratio, was 31.28 where it is preferable to be more than 4^9^. A comparatively low rate of variation coefficient was 6.95% indicated a superior accuracy and reliability of the model experiments. Accordingly, this model could be used to navigate the design space with a reasonable accuracy.

Final equation in terms of coded variables

$$\begin{aligned} {\text{AuNPs synthesis }} & = \, - {2}.{41357 } + { 19}.{89732 }*{\text{ A}} - { 3}.{24619 }*{\text{ B }} + \, 0.{42238 }*{\text{ C}} \\ & \quad + 0.{28167 }*{\text{ D }} + { 5}.{\text{99E}} - 0{3 }*{\text{ E }} - \, 0.0{3317 }*{\text{ F }} + \, 0.{6}0{119 }*{\text{ A }}*{\text{ B}} \\ & \quad \, - { 2}.{59821 }*{\text{ A }}*{\text{ C}} - \, 0.0{1347 }*{\text{ A }}*{\text{ E }} + \, 0.{42333 }*{\text{ B }}*{\text{ C}} \\ \end{aligned}$$

where A: casein hydrolysate; B: dextrin; C: initial pH; D: incubation period; E: inoculum size; F: temperature.

These positive and negative signs in the equation, where for the variable of a positive sign, AuNPs production increases with its increase and vice-versa. On the other hand, the variable of a negative sign, AuNPs production increases with its decrease and vice-versa^29^. Interactions between two variables could appear as a synergistic effect (positive coefficient) or as an antagonistic effect (negative coefficient)^30^.

Among the 6 variables, casein hydrolysate concentration, initial pH, inoculum size and incubation period showed positive signs of the effect on AuNPs synthesis; however other factors showed negative signs of the effect.

The contribution of interaction between casein hydrolysate and initial pH in AuNPs production represents the highest percentage in this model 10.92% followed by interaction between dextrin and initial pH then initial pH and casein hydrolysate with 8.32, 7.05 and 4.37, respectively, as shown in Table 7.

**Table 7:** Percentage contribution of model parameters on AuNPs synthesis.

Term	% Contribution
A-casein hydrolysate	4.38
B-dextrin	0.02
C-initial pH	7.05
D-incubation period	3.93
E-inoculum size	0.00
F-temperature	2.92
AB	1.75
AC	10.92
AD	0.052
AE	2.11
BC	8.32
Curvature	58.35
Lack of fit	0.00
Pure error	0.19

Casein hydrolysate concentration, dextrin concentration, initial pH, incubation period, inoculum size and temperature were chosen for further optimization using Box–Behnken design (BBD). They were found to exhibit a significant effect on AuNPs synthesis at the tested concentration levels.

### Box–Behnken design (BBD) for optimization of AuNPs biosynthesis

**Figure 1:**
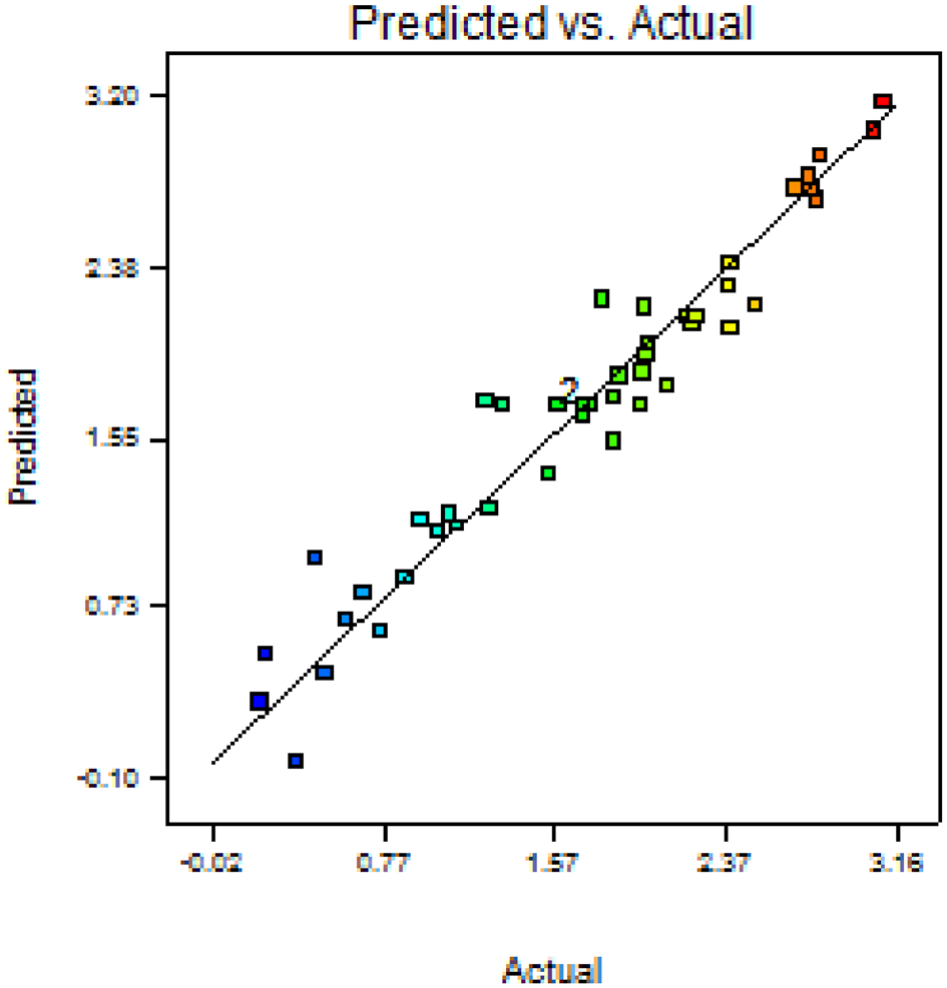
2D plotting of the relation between experimental and predicted results for AuNPs synthesis in Box–Behnken design.

The quadratic BBD experiments were carried out and average values were calculated and recorded. The experimental and predicted results according to BBD were shown in Table 4. Analysis of variance of BBD for AuNPs biosynthesis was shown in Table 8. Analysis of variance of the experimental data obtained by BBD showed that, the model F-value was 14.40 and *p*-value was < 0.0001 which implied a highly significant statistical model. The *p*-values of the model terms indicate significant parameters if they are < 0.05. The significant model terms were: A, C, AB, AC, AD, AE, BC, C^2^, A^2^B, A^2^D, AB^2^, AD^2^, B^2^E and BD^2^. The model "Lack of Fit F-value" of 2.02 implied that the Lack of Fit was not significant relative to the pure error. This could be shown by the relation between expermintal and predicted results (Fig. 1).

**Table 8:** Analysis of variance (ANOVA) of BBD of AuNPs biosynthesis, using the extracellular filtrate of *Bacillus subtilis* NRC1.

Source	Sum of squares	df	Mean square	F-value	p-value* prob > F
Model	26.42	23	1.15	14.40	< 0.0001
A-casein hydrolysate	3.66	1	3.66	45.87	< 0.0001
B-dextrin	0.01	1	0.01	0.18	0.6750
C-intial pH	4.47	1	4.47	56.09	< 0.0001
D-inoculum size	0.13	1	0.13	1.69	0.2077
E-temperature	0.34	1	0.34	4.22	0.0520
AB	0.38	1	0.38	4.82	0.0390
AC	0.59	1	0.59	7.43	0.0123
AD	1.15	1	1.14	14.36	0.0010
AE	0.80	1	0.80	10.04	0.0044
BC	1.58	1	1.58	19.75	0.0002
BD	1E−04	1	1E−04	0.00	0.9721
BE	0.09	1	0.09	1.21	0.2842
A2	0.06	1	0.06	0.71	0.4098
B2	0.01	1	0.01	0.11	0.7424
C2	0.46	1	0.46	5.73	0.0256
D2	0.09	1	0.09	1.12	0.3017
A2B	2.33	1	2.33	29.25	< 0.0001
A2D	1.54	1	1.54	19.28	0.0002
AB2	1.63	1	1.63	20.40	0.0002
AD2	2.70	1	2.70	33.86	< 0.0001
B2E	0.67	1	0.67	8.39	0.0083
BC2	0.06	1	0.06	0.79	0.3837
BD2	2.08	1	2.08	26.09	< 0.0001
Residual	1.75	22	0.08		
Lack of fit	1.53	17	0.09	2.02	0.2241
Pure error	0.22	5	0.05		
Cor total	28.17	45			

F value = 14.40; R^2^ = 0.94; Adjusted R^2^ = 0.87.

*: Values of "prob > F" less than 0.05 indicate model terms are significant.

The model correlation coefficients; R^2^, adjusted- R^2^ and predicted- R^2^ values were 0.94, 0.87 and 0.69, respectively. The adjusted- R^2^ was in a reasonable agreement with the predicted-R^2^ which also indicated a strong relationship between experimental and predicted results. In the present model, the ratio of the model adequate precision which measures the signal to noise ratio was 15.62, indicating that the model generated an adequate signal to design space. The model coefficient of variation value was 16.58%. All the previously mentioned information indicated the accuracy and the reliability of the model and that it could be used to navigate the design space with a reasonable accuracy.

Final equation in terms of coded variables

$$\begin{aligned} {\text{AuNPs synthesis }} & = \, - { 29}.{9612 } + { 3}.{792542 }*{\text{ A }} + { 9}.{9893335 }*{\text{ B }} + { 7}.{2}0{27652 }*{\text{ C}} \\ & \quad + \, 0.0{118294 }*{\text{ D }} + \, 0.{1655952 }*{\text{ E }} + \, 0.{1631236 }*{\text{ A }}*{\text{ B}} - \, 0.{6875 }*{\text{ A }}*{\text{ C }} \\ & \quad + \, 0.0{273124 }*{\text{ A }}*{\text{ D }} + \, 0.{1598214 }*{\text{ A }}*{\text{ E }} - { 1}.{238333 }*{\text{ B }}*{\text{ C }} \\ & \quad - \, 0.0{18244 }*{\text{ B }}*{\text{ D }} - 0.{23}0{667 }*{\text{ B }}*{\text{ E }} - { 8}.{917338 }*{\text{ A}}^{{2}} \\ & \quad - \, 0.{799969 }*{\text{ B}}^{{2}} - \, 0.{516}0{61 }*{\text{ C}}^{{2}} - { 2}.{\text{46E}} - 0{5 }*{\text{ D}}^{{2}} + { 2}.{2959184 }*{\text{ A}}^{{2}} *{\text{ B}} \\ & \quad + \, 0.0{2}0{38}0{6 }*{\text{ A}}^{{2}} *{\text{ D }} - \, 0.{62}00{4 }*{\text{ A }}*{\text{ B}}^{{2}} - \, 0.000{143 }*{\text{ A }}*{\text{ D}}^{{2}} \\ & \quad + \, 0.0{42 }*{\text{ B}}^{{2}} *{\text{ E }} + \, 0.{1183333 }*{\text{ B }}*{\text{ C}}^{{2}} + { 5}.{\text{421E}} - 0{5 }*{\text{ B }}*{\text{ D}}^{{2}} \\ \end{aligned}$$

where A: casein hydrolysate, B: dextrin, C: initial pH, D: inoculum size, E: temperature.

**Figure 2:**
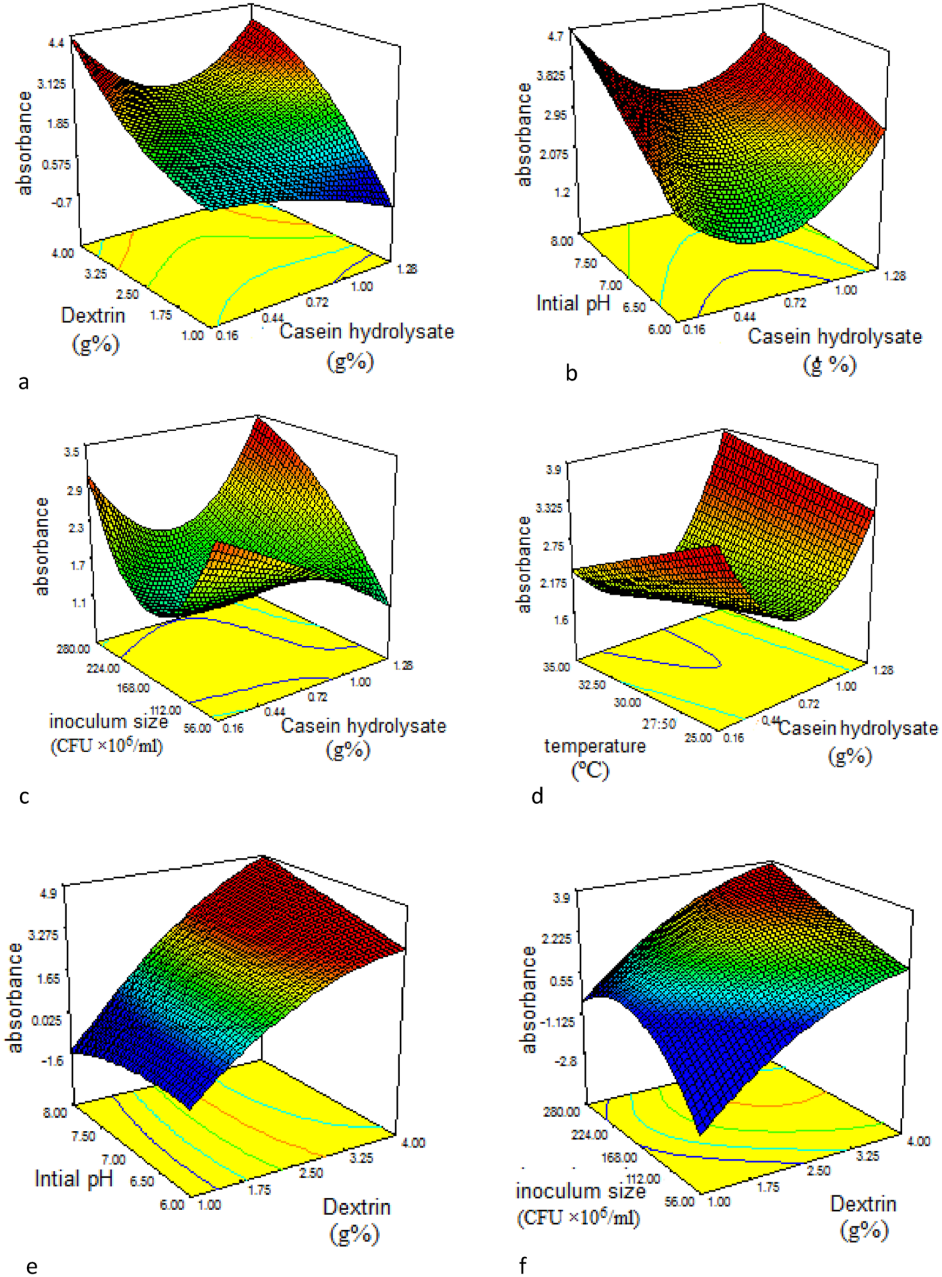

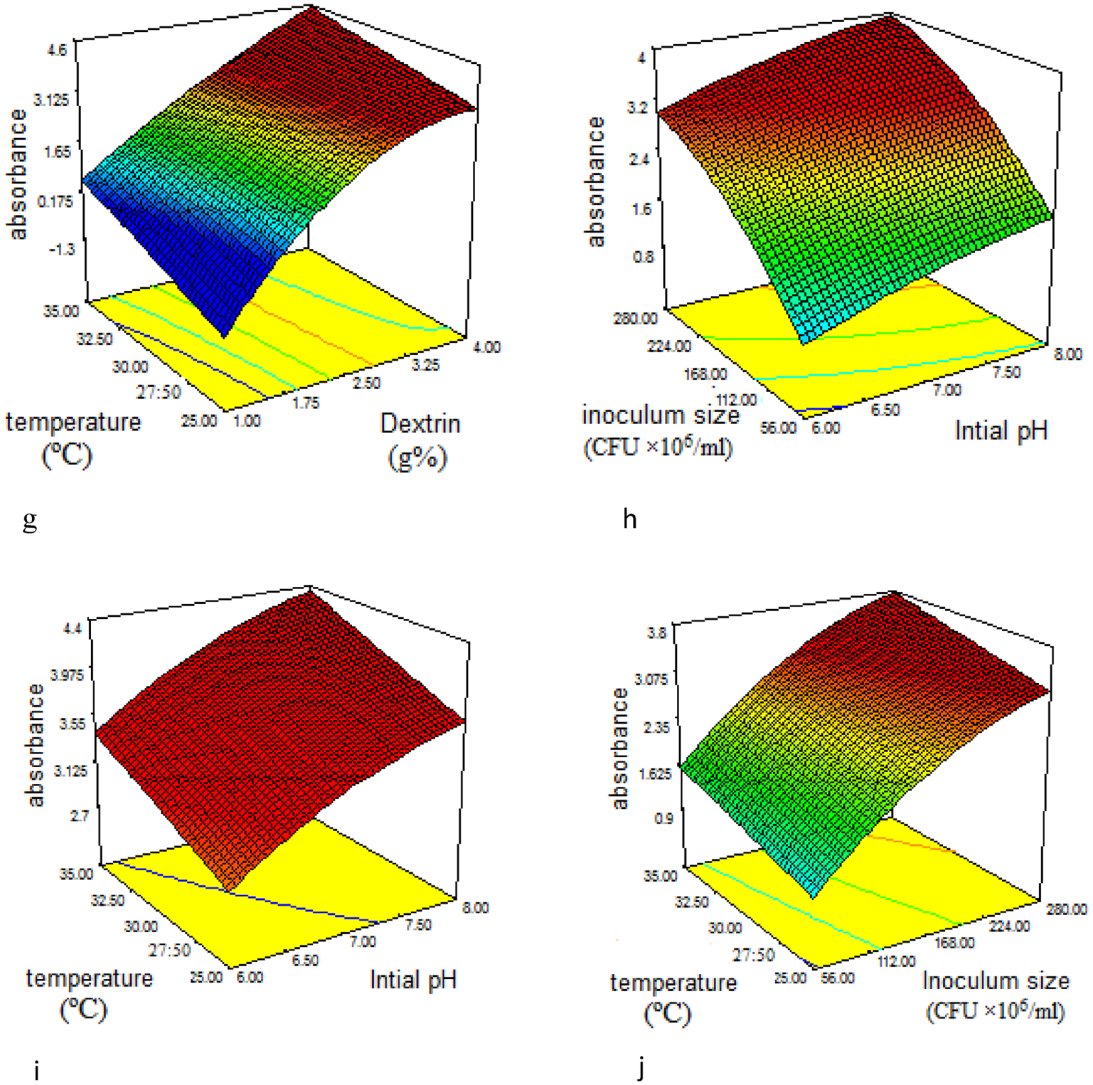
3D response surface plot for the effect of different factors on AuNPs biosynthesis and factor-factor interactions between (**a**) casein hydrolysate and dextrin (**b**) casein hydrolysate and initial pH (**c**) casein hydrolysate and inoculum size (**d**) casein hydrolysate and temperature (**e**) dextrin and initial pH (**f**) dextrin and inoculum size (**g**) dextrin and temperature (**h**) initial pH and inoculum size (**i**) initial pH and temperature (**j**) inoculum size and temperature.

The 3D response surface plots (Fig. 2a-j) were used to demonstrate the relationship between AuNPs absorbance and the experimental levels of each independent variable. Each plot represents the interaction between two variables, whereas the other variables were kept at zero levels. Through simple scanning of the 3D-figures, we could determine the positive and negative effects of different variables.

Figure 2a illustrates the interaction effects between casein hydrolysate and dextrin, whereas it was found that the change in casein hydrolysate concentration showed no marked effect on AuNPs yield. While the increase in dextrin concentration from1%- 4% showed a gradual increase in AuNPs biosynthesis. Figure 2b depicts the interaction between casein hydrolysate and initial pH of fermentation medium. As shown in the figure, the maximum AuNPs synthesis was at the lowest and the highest levels of casein hydrolysate but AuNPs biosynthesis increased with the gradual increase in initial pH reaching maximum at pH 8. Figure 2c showed that maximum AuNPs biosynthesis was at 0.16% casein hydrolysate (the lowest concentration). However, the maximum yield was at the highest and the lowest inoculum size. Figure 2d represents the interaction between casein hydrolysate and temperature, maximum AuNPs biosynthesis was at the highest and the lowest levels of casein hydrolysate. While the change in temperature didn’t affect markedly their yield. As shown in Fig. 2e, by the increase in dextrin concentration, AuNPs production increased. Whereas, the change in the medium initial pH didn’t affect muchly their production. The interaction between dextrin and inoculum size was demonstrated in Fig. 2f, the gradual increase in dextrin concentration led to an increase in AuNPs yield reaching its maximum value at 4% dextrin. While their maximum yield was at an inoculum size near the central point. On the other hand, the increase in both dextrin concentration and temperature led to an increase in AuNPs as shown in Fig. 2g. While in Fig. 2h, the gradual increase in the fermentation medium initial pH slightly affected the nanoparticles productivity. However, the increase in bacterial inoculum size led to a sharp increase in AuNPs biosynthesis. Figure 2i depicts the interaction between initial pH and temperature, the plot reveals that the increase in both factors remarkably increased AuNPs yield. Figure 2j represents the 3D plot as a function of inoculum size and temperature. According to the figure, the change in temperature didn’t affect AuNPs. However, the increase in inoculum size till the highest value (280 CFU × 10^6^/ml) led to maximum AuNPs yield.

### Model validation

The numerical optimization for AuNPs synthesis was attempted with Design expert, producing optimum conditions of 0.74% (w/v) casein hydrolysate, 3.99% (w/v) dextrin, pH 7.76, 47 × 10^6^ CFU/ml inoculum size and 25 $^\circ$C temperature to give the maximum AuNPs biosynthesis of 3.30 absorbance intensity. To substantiate the optimum concentrations, an experiment with the above particularized conditions was carried out and the result was corresponded to 3.25 ± 0.015. The experimental result was found to be very close to the anticipated value (predicted value) which was 3.30. This result showed that the model was highly valid and the obtained data had a confidence factor of 98.48%. This high degree of precision affirmed the reliability of the model. Statistical optimization resulted in a 2.6-fold increase in AuNPs production compared with that of the non-optimized medium. This will be cost effective for synthesis of AuNPs due to the high price of AuNPs precursor.

### Nitrate reductase activity

The nitrate reductase activity was estimated in the extracellular filtrate after the growth of *Bacillus subtilis* NRC1 in this optimized medium. Nitrate reductase was found to be 4.3 µmol/ml/min.

## Discussion

AuNPs have unique optical, electrical and photothermal properties. They have been extensively employed in the biomedical field; in drug delivery, imaging and treatment of major life threatening diseases such as cancer, rheumatoid arthritis, chronic inflammation, pathological neo-vascularization and neoplastic disorders^3,14^. Also, they have many applications in microbiology, medicine, environmental sensors and biosensors^31^.

As a result of the high price of the AuNPs precursor, the productivity of AuNPs must be increased.

RSM is a collection of statistical and mathematical techniques which are very useful for developing, improving and optimizing processes. RSM is a known statistical method for the optimization of the medium components and the growth conditions for the production of NPs^30^. Statistical experimental design methodologies are considered as powerful tools used for the optimization of target bioactive metabolites production. It has the advantages of taking into account the interaction among the factors, less time consuming, limited number of experiments and avoids the erroneous interpretation occurring in one-variable-at-a-time optimization. There are many available experimental designs such as Plackett–Burman (PBD), central composite (CCD) and Box–Behnken (BBD)^32^.

In this study, Plackett–Burman design (PBD) was applied, followed by Box–Behnken design (BBD) for the maximum AuNPs synthesis by *Bacillus subtilis* NRC1.

At the first, PBD was applied to analyze and conclude the most significant factors affecting AuNPs synthesis. The determination coefficient (R^2^) of this model was 0.99, indicating that there was a very good fit between the observed and predicted values of AuNPs biosynthesis and implied that the model is reliable for this process.

Casein hydrolysate concentration, dextrin concentration, initial pH, incubation period, inoculum size and temperature were chosen for further optimization using Box–Behnken design (BBD). They were found to exhibit a significant effect on AuNPs synthesis at the tested concentration levels.

The second-order design BBD plays an important role in response surface methodology especially when the design economy and precise prediction are desired. BBD is a kind of rotatable design which uses just three levels of each factor^18^.

The numerical optimization for AuNPs synthesis was attempted with Design expert, producing optimum conditions of 0.74% (w/v) casein hydrolysate, 3.99% (w/v) dextrin, pH 7.76, 47 × 10^6^ CFU/ml inoculum size and 25 $^\circ$C temperature to give the maximum AuNPs biosynthesis of 3.30 absorbance intensity.

To our knowledge, there are few reports about using RSM for optimization of bacterial fermentation conditions for the biosynthesis of AuNPs.

Manivasagan et al.^33^ studied optimization of fermentation medium composition for maximum amylase production by using *Streptomyces* sp. MBRC-82 for the production of AuNPs using RSM. The variables chosen for PBD were soluble starch, yeast extract, peptone, K_2_HPO_4_, MgSO_4_.7H_2_O and NaCl. The significant factors resulted from the design were introduced in BBD for further optimization studies. They concluded the optimized medium components which were soluble starch (5.84 g/l), peptone (3.51 g/l) and NaCl (0.38 g/l) for maximum amylase production used in synthesis of extracellular AuNPs. While Manivasagan and Oh^34^ used PBD for screening of the significant variables for the production of fucoidanase by green synthesis using* Streptomyces* sp.. The enzyme is then used in the biosynthesis of AuNPs. Among the variables studied, the most significant variables identified as influencing the fucoidanase production were wheat bran, kelp powder and NaCl. They were selected and their optimal levels were identified using BBD. Maximum fucoidanase production could be achieved when the wheat bran, kelp powder and NaCl were at 3.34 g/l, 0.70 g/l, and 0.88 g/l, respectively. This optimization led to an enhancement of fucoidanase production from 3.71 U/ml to 16.74 U/ml, which was further used in AuNPs biosynthesis.

It was reported that Rane et al.^35^ carried out optimization of the production of biosurfactant by *Bacillus subtilis* ANR88 for application in AgNPs and AuNPs synthesis, using PBD. They selected 11 variables and it was found that only 4 variables significantly affected the culture growth which were ammonium ferric citrate, molasses, pH and inoculum size for biosurfactant production for AgNPs and AuNPs synthesis. Also, it was found that ammonium ferric citrate, molasses and pH showed a positive correlation with the biosurfactant production, while the inoculum size was found to have a negative effect on biosurfactant production. Also, the maximum biosurfactant yield was in the medium containing 4% molasses, 0.25% ammonium ferric citrate and pH 7. The produced biosurfactant efficiently used for synthesis almost uniform (in size and shape) AgNPs and AuNPs. Recently, Singh et al.^14^ used CCD to increase nitrate reductase activity through optimization of *Bacillus licheniformis* growth for using it in AuNPs biosynthesis. Maximum AuNPs biosynthesis was obtained using the optimized medium variables which contained 2.1 g/l glucose, 14.05 g/l peptone, 4.14 g/l yeast extract and 3.91 g/l KNO_3_.

Honary et al.^36^ studied the biosynthesis and mathematical optimization process for iron oxide NPs produced by *Penicillium waksmanii*s useing Box–Behnken experimental design to investigate the effects of different factors such as pH, temperature and FeCl_3_ concentration. The results showed R^2^ value of 0.9992 which indicated the accuracy and ability of the polynomial model. In the study of Barabadi et al.^37^ for biosynthesis optimization of AgNPs produced by *Penicillium citrinum* using BBD, the effective factors were AgNO_3_ (mM) concentration, solution pH, incubation temperature (°C), shaking rate (rpm) and incubation time (hours). They found that R^2^ value of the obtained model was 0.8894, indicating a good correlation between both observed and predicted values. Thus, they concluded the optimum conditions for the biosynthesis of AgNPs by *Penicillium citrinum* with a low cost and time.

In this study the nitrate reductase activity in extracellular filtrate of *Bacillus subtilis* NRC1 was found to be 4.3 µmol/ml/min. It was reported the important role of this enzyme in reduction of HAuCl_4_ into nanogold and their stabilization^3,6,25,38^.

## Data Availability

All data generated or analysed during this study are included in this published article.
